# Triterpenoid Saponin AG8 from *Ardisia gigantifolia* stapf. Induces Triple Negative Breast Cancer Cells Apoptosis through Oxidative Stress Pathway

**DOI:** 10.1155/2020/7963212

**Published:** 2020-10-13

**Authors:** Li-Hua Mu, Li-Hua Wang, Teng-Fei Yu, Yu-Ning Wang, Hong Yan, Ping Liu, Can Yan

**Affiliations:** ^1^Department of Clinical Pharmacology, The Medical Supplies Center, Chinese PLA General Hospital, Beijing 100853, China; ^2^Department of Ultrasound, Beijing Tiantan Hospital of Capital Medical University, Beijing 100050, China; ^3^Clinical Surgery Division, The First Medical Center, Chinese PLA General Hospital, Beijing 100853, China; ^4^Department of Obstetrics and Gynecology, The First Medical Center, Chinese PLA General Hospital, Beijing 100853, China; ^5^Department of Basic Theory of Chinese Medicine, School of Pre-Clinical Medicine, Guangzhou University of Chinese Medicine, Guangzhou 510060, China; ^6^The Research Centre of Basic Integrative Medicine, Guangzhou University of Chinese Medicine, Guangzhou 510060, China

## Abstract

Triple-negative breast cancers (TNBCs) are associated with poor patient survival because of the absence of estrogen receptor (ER), progesterone receptor (PR), and human epidermal growth factor receptor 2 (HER2) expressions. Our previous studies have shown that the triterpenoid saponin AG8 from *Ardisia gigantifolia* stapf. inhibits the proliferation of MDA-MB-231 cells. In this study, the effects of AG8 were further analyzed in different TNBC cell types: MDA-MB-231, BT-549, and MDA-MB-157 cells. AG8 inhibited the viability of MDA-MB-231, BT-549, and MDA-MB-157 cells in a dose-dependent manner and showed stronger cytotoxicity to African American (AA) and mesenchymal (M) subtypes than Caucasian (CA) and mesenchymal stem-like (MSL) subtypes, respectively. AG8 impaired the uptake of MitoTracker Red CMXRos by the mitochondria of TNBC cells in a dose-dependent manner, and this was recovered by *N*-acetyl-l-cysteine (NAC). AG8 affected GSH, SOD, and MDA levels of TNBC cells, but different TNBC subtypes had different sensitivities to AG8 and NAC. In addition, we found that AG8 increased the Bax/Bcl-2 ratio and the levels of cytoplasmic cytochrome c and significantly decreased phosphorylation of ERK and AKT in BT549 and MDA-MB-157 cells. AG8 elicited its anticancer effects through ROS generation, ERK and AKT activation, and by triggering mitochondrial apoptotic pathways in TNBC cells. AG8 had selective cytotoxic effects against the AA and M TNBC subtypes and markedly induced MDA-MB-157 (AA subtype) cell apoptosis through pathways that were not associated with ROS, which was different from the other two subtypes. The underlying mechanisms should be further investigated.

## 1. Introduction

Triple negative breast cancers (TNBCs) lack estrogen receptor (ER), progesterone receptor (PR), and human epidermal growth factor receptor 2 (HER2) expression and account for approximately 15-25% of total breast cancer cases [1]. TNBCs are highly recurrent and metastatic with poor prognosis and often occur in young patients [2, 3]. Common hormone therapy or targeted therapies are inefficient for TNBCs because of the deficiencies in necessary receptors, and therefore, combination chemotherapy treatments are usually given to TNBC patients; however, drug resistance and adverse side effects occur frequently [4, 5]. TNBC is a very heterogeneous disease with at least six molecular subtypes: immunomodulatory (IM), mesenchymal (M), mesenchymal stem-like (MSL), basal-like 1 and 2 (BL1 and BL2), and luminal androgen receptor (LAR) [6]. TNBC can also be divided according to race into African American (AA) type and Caucasian (CA) type [7]. These TNBC subtypes have different responses to standard of care (SOC) treatments, because each of them has its own unique ontology [8]. Since most currently available therapies cannot benefit TNBCs, therapeutic agents with minimal toxicities and better efficacy need to be developed.

According to our previous studies, triterpenoid saponins from *Ardisia gigantifolia* stapf. showed cytotoxic activities towards several kinds of cancer cells [9–11]. Some of the triterpenoid saponins showed prominent cytotoxicity against breast cancer cells [12, 13]. Among them, triterpenoid saponin AG8 isolated from *A. gigantifolia* stapf. inhibited proliferation of MDA-MB-231 cells. In this study, AG8 was selected for further analysis on different TNBC cell types including MDA-MB-231, BT-549, and MDA-MB-157. This study is aimed at investigating the effects of AG8 on the oxidative stress pathway and cell death induction in TNBC cells.

## 2. Materials and Methods

### 2.1. Chemicals and Reagents

**Figure 1:**
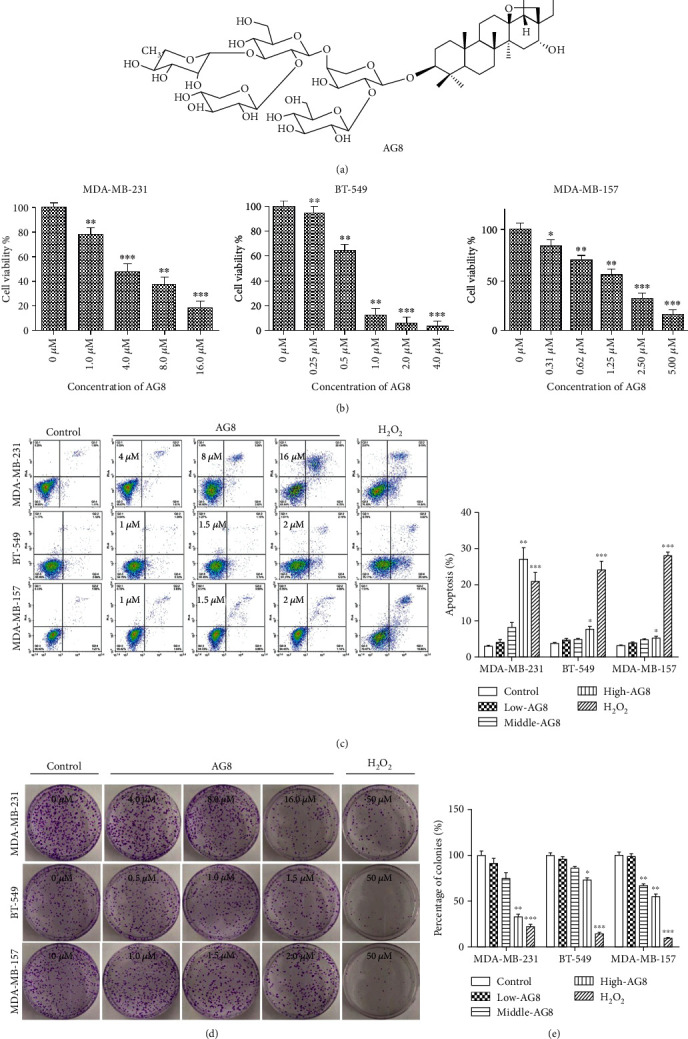
Effects of AG8 on TNBC cells. **(**a) Structure of AG8. (b) AG8 dose-dependently reduces the viability of MDA-MB-231, BT-549, and MDA-MB-157 cells. (c) Cells were flow cytometrically analyzed after staining with an Annexin V-FITC kit. (d) Representative images of colony formation of differently treated TNBC cells were shown. (e) Quantitative results of colony formation assays were displayed. Data were represented as mean as mean ± SD from three independent experiments, ^∗^*P* < 0.05, ^∗∗^*P* < 0.01, and ^∗∗∗^*P* < 0.001 versus control.

AG8 (Figure 1(a), purity: >95%) was isolated from *A. gigantifolia* stapf. as previously described [10]. The 3-(4,5-dimethylthiazol-2-yl)-2,5-diphenyltetrazolium bromide (MTT) was purchased from Sigma-Aldrich (St. Louis, MO, USA).

### 2.2. Cell Culture

The TNBC cell lines MDA-MB-231, MDA-MB-157, and BT-549 were purchased from Cell Culture Collection of the Chinese Academy of Medical Sciences (Beijing, China). MDA-MB-231 and MDA-MB-157 cells were cultured in L-15 medium (Gibco, Grand Island, NY, USA) supplemented with 10% fetal bovine serum (FBS) and 1% penicillin/streptomycin with 5% CO_2_ at 37°C in a humidified atmosphere. BT-549 cells were grown in DMEM medium (Gibco) containing 20% FBS at 37°C in a humidified atmosphere of 5% CO_2_.

### 2.3. Cell Viability Assay

The effects of AG8 on cell viability were evaluated using the MTT assay. To this end, MDA-MB-231, MDA-MB-157, and BT-549 cells were treated with increasing concentrations of AG8 for 24 h. Then, the cells were incubated with MTT solution for 4 h, the supernatant was aspirated, and the formazan crystals were dissolved using DMSO. Finally, the cell viability was calculated by measuring the absorbance at 570 nm using a microplate reader (Perkin-Elmer, Inc., 1420-012, Shanghai, China). The antioxidant *N*-acetyl-l-cysteine (NAC; Sigma, # A7250) in PBS (final concentration 4 mM) was added to the cells for 1 h before the addition of AG8, and then, the cells were cultured with NAC and AG8 for 24 h before assaying for cell viability.

### 2.4. Proliferation and Clonogenicity Assay

For the assessment of proliferation, cells were seeded into 6-cm culture dishes and counted after 24 h and treated by different concentrations of AG8 for 24 h and replaced with regular culture to continuously incubate for 14 days. Colonies were washed and fixed with 4% paraformaldehyde for 15 min. Then, colonies were stained with 0.1% crystal violet for 10 min and counted.

### 2.5. Measurement of Cell Apoptosis

Apoptosis of TNBC cells was examined using Annexin V-FITC and PI double staining. The cells were treated with different concentrations of AG8 for 24 h: MDA-MB-231 cells with 0, 4.0, 8.0, and 16.0 *μ*M AG8; MDA-MB-157 with 0, 1.0, 1.5, and 2.0 *μ*M AG8; and BT-549 cells with 0, 0.5, 1.0, and 1.5 *μ*M AG8. Then, the cells were collected, washed with PBS, and stained by Annexin V-FITC kit (Keygen, Jiangsu, China). Cell apoptosis was analyzed using flow cytometry (FACS Calibur; Becton Dickinson, San Jose, CA, USA). NAC (4 mM) was added to the TNBC cells 1 h before treatment with AG8, and then, the cells were cultured with NAC and AG8 for 24 h before the measurement of cell apoptosis.

### 2.6. Measurement of Mitochondria Activity

MDA-MB-231, MDA-MB-157, and BT-549 cells were incubated with different concentrations of AG8 for 24 h, and then, 100 nM MitoTracker Red CMXRos was added to the cells (Keygen, Jiangsu, China). After incubation at 37°C for 30 min, the cells were observed by using a fluorescence microscope (Olympus BX60, Tokyo, Japan). NAC (4 mM) was added to the TNBC cells 1 h before AG8 treatment, and then, the cells were cultured with NAC and AG8 for 24 h before the measurement of mitochondria activity.

### 2.7. Measurement of Intracellular Levels of ROS

Intracellular ROS was estimated using the fluorescent probe, 2′,7′-dichlorofluorescein diacetate (DCFH-DA). Briefly, the cells were treated with different concentrations of AG8 with or without NAC (4 mM) for 24 h. MDA-MB-231, MDA-MB-157, and BT-549 cells (0.5 × 10^5^ cells/ml) were then incubated in a culture medium containing 10 *μ*M DCFH-DA for 20 min at 37°C. Following incubation, cells were washed with PBS and resuspended in PBS for ROS accumulation measurement using flow cytometry.

### 2.8. Measurement of SOD, GSH, and MDA Levels

After treatment with AG8 for 24 h, the superoxide dismutase (SOD), glutathione (GSH), and malondialdehyde (MDA) levels in MDA-MB-231 cells (AG8: 0, 4.0, 8.0, and 16.0 *μ*M), MDA-MB-157(AG8: 0, 1.0, 1.5, and 2.0 *μ*M), and BT-549 cells (AG8: 0, 0.5, 1.0, and 1.5 *μ*M) were analyzed using the SOD WST-1 assay kit, GSH assay kit, and MDA assay kit from Nanjing Jiancheng Bio-Engineering Institute (Nanjing, Jiangsu, China, #A001-3, #A006-2 and #A003-4) according to the manufacturer's instructions. NAC (final concentration 4 mM) was added to the TNBC cells 1 h before AG8 treatment, and then, the cells were cultured with NAC and AG8 for 24 h before the measurement of cell apoptosis.

### 2.9. Western Blot Analysis

BT549 and MDA-MB-157 cells were treated with different concentrations of AG8 (1.0 and 1.5 *μ*M) with or without NAC (4 mM) for 24 h. The TNBC cells were collected and lysed in total protein extraction reagent with proteinase inhibitors. The protein concentrations were measured by the BCA protein assay kit. Protein samples from treated TNBC cells were separated by SDS–PAGE and then transferred onto PVDF membranes, which were washed and blocked in 5% nonfat dry milk in TBST for 1 h at 25°C. Subsequently, the membranes were washed and incubated with indicated primary antibodies against cleaved-caspase-3, Bax, Bcl-2, cytochrome C, AKT, pAKT, ERK, and pERK (Cell Signaling Technology, MA, USA) at 4°C overnight and further incubated with horseradish peroxidase-conjugated secondary antibodies at 25°C for 1 h. The load protein bands were visualized using the enhanced chemiluminescent detection reagent (Pierce, Rockford, IL, USA).

### 2.10. Statistical Analysis

All data were presented as means ± standard deviation (SD) from three independent experiments. Data were analyzed by ANOVA. Statistical comparisons were evaluated using Student's *t*-test. *P* values of less than 0.05 were considered to be statistically significant.

## 3. Results

### 3.1. AG8 Inhibited Cell Proliferation and Growth of Triple Negative Breast Cancer Cells

To determine whether AG8 differentially affects TNBC cell proliferation, its effects on MDA-MB-231 (MSL, CA), BT-549 (M, CA), and MDA-MB-157 (MSL, AA) were investigated. As shown in Figure 1(b), AG8 inhibited the viability of MDA-MB-231, BT-549, and MDA-MB-157 cells in a dose-dependent manner, and the IC_50_ values were 3.80, 0.73, and 1.38 *μ*M, respectively. AG8 showed stronger cytotoxicity in MDA-MB-157 than MDA-MB-231cells, indicating that the AA may be more sensitive to AG8 than CA TNBC cell lines. MDA-MB-231 and BT-549 are CA type TNBCs; AG8 showed increased cytotoxicity to BT-549 (IC_50_ 0.73 *μ*M) compared to MDA-MB-231(IC_50_ 3.80 *μ*M), which means AG8 could have more effects on M subtypes than on MSL subtypes.

To determine the effects of AG8 on cell apoptosis, the apoptosis of MDA-MB-231, BT-549, and MDA-MB-157 cells were treated with AG8 for 24 h and double stained with annexin-V/PI in order to assay for apoptosis using flow cytometry. As shown in Figure 1(c), after treatment with 0, 4.0, 8.0, and 16.0 *μ*M AG8 for 24 h, the percentage of apoptotic cells in MDA-MB-231, BT-549, and MDA-MB-157 cells was increased significantly. Incubation of MDA-MB-231 cells with high doses of AG8 resulted in higher levels of apoptosis than H2O2 which was used as positive control. These results indicated that AG8 could reduce cell viability by increasing the number of early or late apoptotic cells in a dose-dependent manner. The colony formation assay indicated that AG8 decreases the percentage of colonies in a dose-dependent manner, but this effect was lower than that of H2O2 (Figures 1(d) and 1(e)). Taken together, all the above results indicated that AG8 inhibited TNBC cell proliferation and caused apoptosis *in vitro*.

### 3.2. *N*-Acetyl-Cysteine Restored Viability of AG8-Treated TNBC Cells

**Figure 2:**
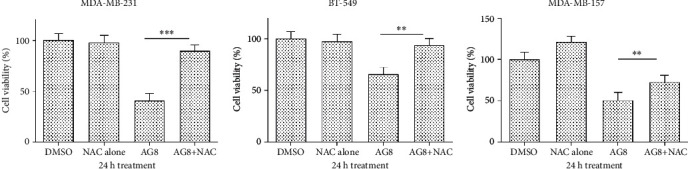
Effects of NAC on the viability of AG8 against TNBC cells. MDA-MB-231, BT-549, and MDA-MB-157 cells were seeded in 6-well plates at a density of 100,000 cells per well and treated 24 h later with AG8 or vehicle control (DMSO) ± NAC (4 mM) for 24 h. Cell viability was assessed by the MTT assay and expressed as % optical density relative to that in DMSO-treated cells. Data (*n* = 3) are expressed as mean ± SD from three independent experiments, ^∗∗^*P* < 0.01 and ^∗∗∗^*P* < 0.001 versus AG8.

To examine the effects of the antioxidant *N*-acetyl-l-cysteine (NAC) on the viability of AG8-treated TNBC cells, MDA-MB-231(AG8, 4.0 *μ*M), BT-549 (AG8, 1.0 *μ*M), and MDA-MB-157(AG8, 1.5 *μ*M) cells were pretreated with NAC (4 mM) prior to 24 h incubation with AG8. As shown in Figure 2, NAC alone had no effect on the viability of MDA-MB-231 and BT-549 cells, while increased the viability of MDA-MB-157 cells. Following treatment with AG8, viability of MDA-MB-231, BT-549, and MDA-MB-157 cells was dramatically decreased to 40.3%, 65.3%, and 50.3%, respectively, and in the AG8+NAC group, cell viability was significantly increased to 89.3%, 93.6%, and 72.6%, respectively. These results suggested that NAC could significantly counteract the negative effects of AG8 on TNBC cell viability (Figure 2).

### 3.3. AG8 Affected Mitochondrial Functions in TNBC Cells

**Figure 3:**
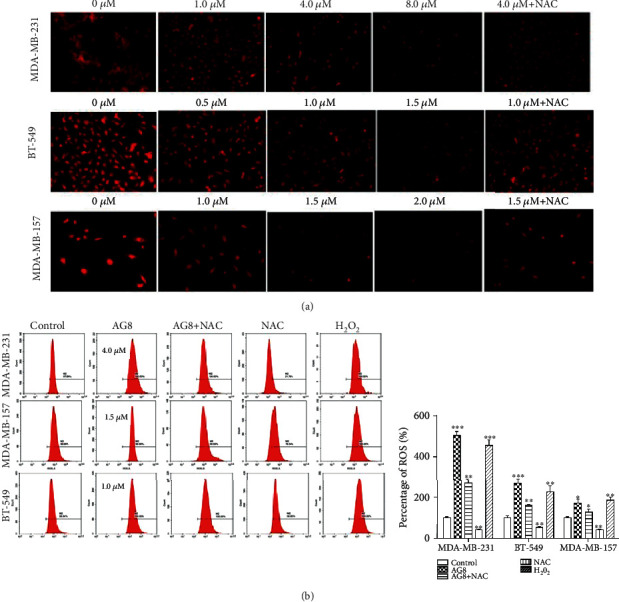
Effects of AG8 on mitochondrial function in TNBC cells. (a) MDA-MB-231, BT-549, and MDA-MB-157 cells were incubated with 0.1% DMSO or with AG8 for 24 h. Following treatment, cells were incubated with 100 nM MitoTracker Red CMXRos for 30 min. The cells were observed by fluorescence microscope (100x). (b) Intracellular ROS generation induced by AG8 and NAC was measured by DCFH-DA (10 *μ*M) and flow cytometry. All results are representative of three independent experiments, ^∗^*P* < 0.05, ^∗∗^*P* < 0.01, and ^∗∗∗^*P* < 0.001 versus control.

It is known that mitochondria dysfunction is one of the many causes of apoptosis. Thus, we examined the effects of AG8 on the mitochondria activity of TNBC cells using MitoTracker Red CMXRos dye. Before the treatment with AG8, MDA-MB-231, BT-549, and MDA-MB-157 cells had many bright red, dot-like fluorescence structures in the cytoplasm of cells (Figure 3(a)). After AG8 treatment for 24 h; the red fluorescence in the cytoplasm was decreased significantly in a dose-dependent manner suggesting that AG8 impaired MitoTracker Red CMXRos uptake by mitochondria. In the AG8+NAC group, the red fluorescence increased dramatically compared to the AG8-alone group at the same concentration (Figure 3(a)). These results clearly demonstrated that the severely impaired function of the mitochondria following treatment with AG8 can be recovered by treating TNBC cells with NAC. Oxidative reactions in mitochondria result in the ROS generation, which are converted to H2O2 by superoxide dismutase. As shown in Figure 3(b), AG8 significantly increased the levels of intracellular ROS in the three TNBC cells compared to the control group, and the antioxidant NAC significantly decreased the levels of intracellular ROS. Upon pretreatment with NAC, the AG8-mediated ROS was significantly inhibited. In MDA-MB-231 and BT-549 cells, AG8 even showed better activity than the positive control H2O2.

### 3.4. Effects of AG8 on GSH, SOD, and MDA Levels in TNBC Cells

**Figure 4:**
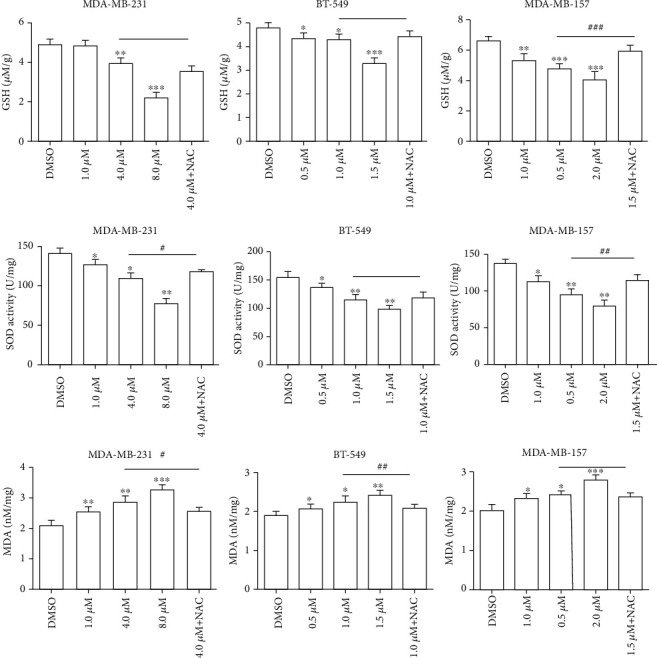
Effects of AG8 on the GSH, SOD, and MDA levels in TNBC cells. Cells were treated with different concentrations of AG8 for 24 h. The images shown are representative results of three biological replicates. Data are expressed as mean ± SD from three independent experiments, ^∗^*P* < 0.05, ^∗∗^*P* < 0.01, and ^∗∗∗^*P* < 0.001 versus DMSO; ^#^*P* < 0.05 and ^##^*P* < 0.01 versus AG8.

To determine the potential effects of AG8 on oxidative stress, the levels of GSH, SOD, and MDA in TNBC cells treated with or without AG8 were further measured. As shown in Figure 4, after incubation with AG8 for 24 h, the levels of GSH and SOD in MDA-MB-231, BT-549, and MDA-MB-157 cells were decreased in a dose-dependent manner, and the levels of MDA were increased in dose-dependent manner. The effects of antioxidant NAC on the GSH, SOD, and MDA levels of AG8-treated TNBC cells were also examined. In MDA-MB-157 cells, the levels of GSH and SOD in the AG8+NAC group (1.5 *μ*M + 4 mM) were increased, and the MDA levels were decreased significantly compared with those in the AG8-alone group at the same concentration. Regarding MDA-MB-231 cells, NAC showed no significant effects on GSH levels, but the SOD and MDA levels were increased and decreased compared with the AG8-alone group at the same concentration, respectively. In BT-549 cells, compared with the 1.0 *μ*M AG8 group, NAC showed significant effects only on MDA levels. In summary, AG8 showed cytotoxicity against TNBC cells by affecting the levels of GSH, SOD, and MDA that are associated with oxidative stress pathway, but different TNBC subtypes had different sensitivities to AG8 and NAC.

### 3.5. Effect of AG8 on the Mitochondria-Dependent Apoptosis Pathway in TNBC Cells

**Figure 5:**
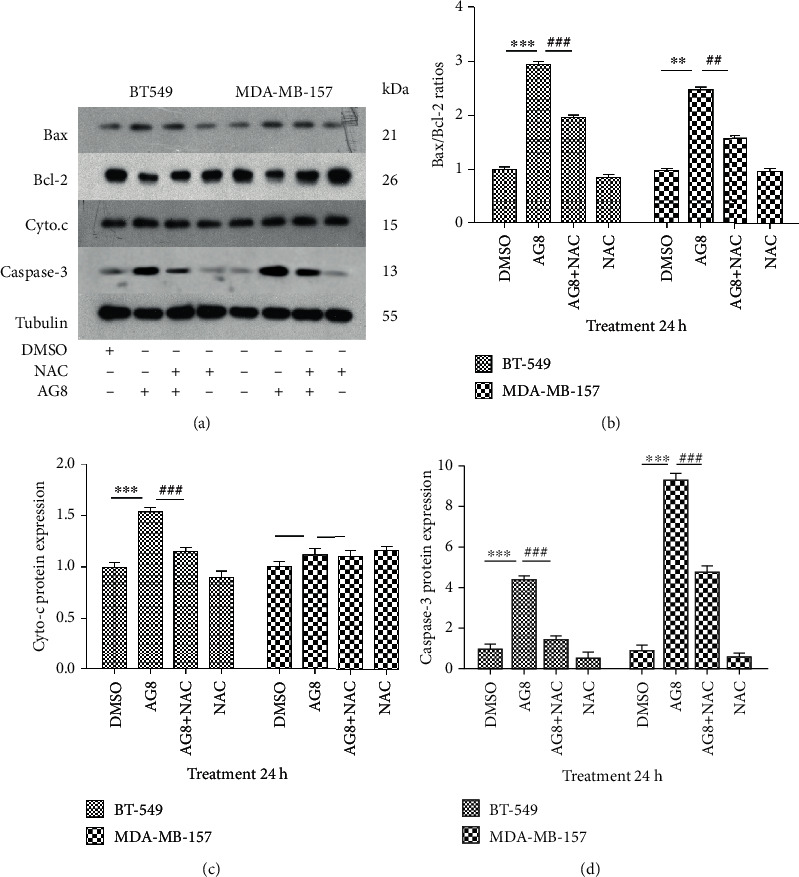
Effect of AG8 on Cyto.c and Bcl-2 family protein expression of TNBC cells. (a) BT549 and MDAMB-157 cells were treated with AG8 (1.0 and 1.5 *μ*M) with or without NAC (4 mM) for 24 h, and protein expression was analyzed using Western blotting. (b) The Bax/Bcl-2 ratio, (c) cycto.c data, and (d) caspase-3 were presented as mean ± SD of three independent experiments. ^∗∗^*P* < 0.01 and ^∗∗∗^*P* < 0.001 compared to the DMSO group; ^##^*P* < 0.01 and ^###^*P* < 0.001 compared to the AG8 group.

After treated with AG8, NAC, or AG8+NAC for 24 h, the expressions of Bax, Bcl-2, and cytochrome c proteins in BT549 and MDA-MB-157 cells were assayed by Western blotting. As shown in Figure 5(a) in BT549, AG8 significantly increased cytochrome c expression, and this effect was counteracted by NAC. However, in MDA-MB-157 cells (AA subtype), AG8 only slightly increased the release of cytochrome c, and NAC also showed a weak effect on this increase (Figures 5(a) and 5(c)). In both BT549 and MDA-MB-157 cells, AG8 significantly increased and decreased the expression of Bax and Bcl-2, respectively, and NAC rescued their levels. For both TNBC cells, AG8 treatment increased the Bax/Bcl-2 ratio significantly (Figure 5(b)), suggesting that the Bcl-2 family of proteins is involved in AG8-induced apoptosis in breast cancer cells. AG8 significantly increased the expression levels of caspase-3 in BT549 and MDA-MB-157 cells, and pretreatment with NAC apparently blocked this effect (Figure 5(d)). These findings suggest that AG8 could induce apoptosis of BT549 and MDA-MB-157 cells through the mitochondria-dependent pathway.

### 3.6. Effects of AG8 on MAPK and AKT Signaling Pathways in TNBC Cells

**Figure 6:**
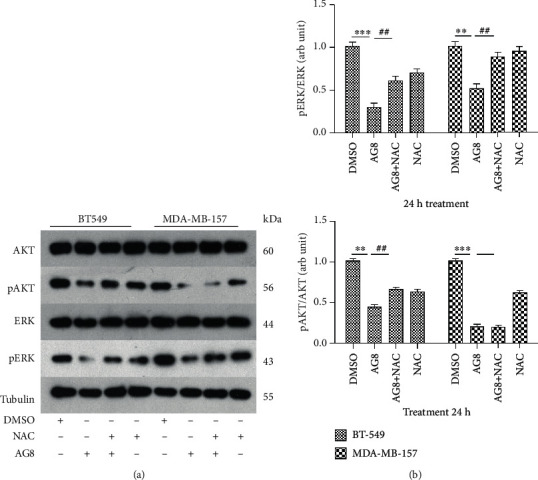
Effects of AG8 on phosphorylation of ERK and AKT in TNBC Cells. (a) BT549 and MDAMB-157 cells were treated with AG8 (1.0 and 1.5 *μ*M) with or without NAC (4 mM) for 24 h, and protein expression was analyzed using Western blotting. (b) Volume intensity of band relative to matched total AKT and ERK band. Data were presented as mean ± SD of three independent experiments. ^∗∗^*P* < 0.01 and ^∗∗∗^*P* < 0.001 compared to the DMSO group; ^##^*P* < 0.01 and ^###^*P* < 0.001 compared to the AG8 group.

We examined the modulatory effects of AG8 on pAKT and pERK in TNBC cells and found that after 24 h of AG8 treatment, the expression of pAKT and pERK in BT549 and MDA-MB-157 cells decreased significantly (Figure 6(a)). In BT549, pretreatment with (4 mM NAC) prior to AG8 treatment, significantly restored the levels of pAKT and pERK, was restored compared with those in the AG8 group (Figure 6(b)). In MDA-MB-157 cells, NAC restored the AG8-induced decrease in pERK without affecting the decrease in pAKT (Figure 6(b)). These findings demonstrated that AG8-induced ROS production may activate MAPK and AKT activation and triggers mitochondrial apoptotic pathways in TNBC cells.

## 4. Discussion

Triple-negative breast cancers (TNBCs) account for 12–20% of all diagnosed breast cancers and have poor overall patient survival [14–16]. The treatment options for TBNC are limited to surgery, radiation, or conventional chemotherapy because few targeted therapies are available [15]. TNBC is a very heterogeneous disease and can be divided into different molecular subtypes: immunomodulatory (IM), mesenchymal (M), mesenchymal stem-like (MSL), basal-like 1 and 2 (BL1 and BL2), and luminal androgen receptor (LAR) or African American (AA) type and Caucasian (CA) type [6, 7]. Each TNBC subtype has its own unique ontology and responds differently to standard of care (SOC) treatments [8]. Here, we investigated the inhibitory effects of AG8, a natural triterpenoid saponin from *Ardisia gigantifolia* stapf., on different TNBC subtypes, namely, MDA-MB-231 (MSL, CA), BT-549 (M, CA), and MDA-MB-157 (MSL, AA).

Apoptosis is a kind of cell death that is genetically controlled, and apoptosis induction is explored as a therapeutic approach for cancer [17, 18]. We found that, regarding MSL TNBC cell lines, AG8 showed stronger cytotoxicity to the AA subtype (MDA-MB-157) than CA (MDA-MB-231). For CA type TNBCs, AG8 showed much more cytotoxicity to M subtypes (BT-549) than to MSL subtype (MDA-MB-231). These results indicating that AG8 may have selective cytotoxicity against AA and M TNBC subtypes need to be confirmed in future studies. Furthermore, our results indicated that the number of early or late apoptotic cells was dose-dependently increased by AG8.

ROS contain unpaired electrons and are generated by partial oxygen reduction [19]. Overproduction of ROS can further cause instability of lipids, proteins, and DNA [20, 21]. ROS accumulation is more harmful to cancer cells because the levels of oxidative stress in cancer cells are higher than that in normal cells. Some triterpenoid saponins have been reported to induce apoptosis via ROS accumulation in cancer cells [22–25]. Therefore, antioxidants (NAC) are often used to promote oxygen reduction. Strikingly, pretreatment with NAC reduced the AG8-induced cytotoxicity, demonstrating that the production of ROS had a role in AG8-induced TNBC cells' apoptosis. In the CA subtype of TNBC cells (MDA-MB-231 and BT-549), cellular activities were recovered similar to those of the DMSO group, while in cells pretreated with NAC, the cellular activity was still significantly decreased compared with that of the DMSO group. These results suggest that AG8 may induce AA subtype TNBC cell apoptosis through not only ROS pathways.

Mitochondrial depolarization is one of the major causes of mitochondrial dysfunction, and mitochondrial dysfunction is critical for cell apoptosis. Impairment of mitochondria membrane integrity will cause depolarization of mitochondrial membrane and finally mitochondria-mediated apoptosis [26–29]. The integrity was examined using MitoTracker Red CMXRos dye [30]. Treatment of cells with AG8 resulted in a dose-dependent damage of the mitochondria membrane, and this effect was reversed by NAC in TNBC cells.

Cellular redox homeostasis is maintained by the balance between antioxidants and prooxidants. Intracellular SOD and GSH are critical antioxidants that protect the cellular components from oxidative damages [31, 32]. While it is produced under oxidative stress and lipid peroxidation, MDA can reflect oxidative damage to the plasma membrane [33]. To further evaluate the effects of AG8 on oxidative stress, we analyzed for the levels of these important cellular antioxidative and prooxidative enzymes in AG8 stimulated TNBC cells. We found that AG8 could significantly decrease the levels of antioxidants SOD and GSH, suggesting that it may initiate redox imbalance in TNBC cells and subsequently induce apoptosis. Our study showed that AG8 significantly increased the levels of the prooxidative enzyme MDA in TNBC cells. It was demonstrated that AG8 might be an appropriate candidate for TNBC treatment because it induces oxidative stress.

Bax and Bcl-2 play crucial roles in cell apoptosis [34]. The mitochondria could initiate an apoptotic cell death pathway through cytochrome c release [35]. The balance of pro- and antiapoptotic members of the Bcl-2 family determines the ultimate apoptosis/survival fate of cells [34]. When the Bax/Bcl-2 ratio is increased, the mitochondrial membrane potential is lost, cytochrome c is released, and subsequently, caspase-9 is activated, resulting in the activation of caspase-3 [35, 36]. Caspase-3 can lead to cell death characterized by cell membrane blebbing, destruction of cell structure, and DNA fragmentation [37]. In AG8-treated BT-549 and MDA-MB-157 cells, the expressions of Bax and Bcl-2 were significantly increased and decreased, respectively, and the increased Bax/Bcl-2 ratio was significantly reduced by NAC treatment. AG8 could induce cytochrome c release from the mitochondria into the cytoplasm, and this effect was also counteracted by NAC. This indicated that AG8-induced apoptosis occurs possibly via the mitochondrial pathway.

The AKT and MAPK signaling pathways are critical pathways involved in the regulation of processes including cell growth, proliferation, differentiation, and apoptosis [38, 39]. Phosphorylated AKT and/or ERK can increase cell proliferation and inhibit apoptosis [40–42]. ROS have been reported to induce apoptosis of tumor cells by activating caspases, MAPK, and PI3K/AKT signaling pathways [43, 44]. The activation of AKT and/or ERK may be a key mechanism for cancer treatment [45]. In addition, AKT may regulate cell apoptosis through modulation of Bcl-2 family members [46]. Therefore, we investigated the cellular signaling pathways involved in AG8-induced apoptosis. We found that AG8 significantly decreased phosphorylation of ERK and AKT in BT549 and MDA-MB-157 cells. NAC could significantly restore the levels of phosphorylated ERK in both two cells, while it only reversed the dramatic decrease in pAKT levels in BT549 cells and had no effects on the decrease of pAKT in MDA-MB-157 cells. These results indicated that ROS may have little effects on the AG8-induced decease of pAKT in MDA-MB-157 (AA subtype), and these different reactions between different subtypes need to be further examined. Therefore, AG8 may interfere with ERK and AKT pathways and triggers mitochondrial apoptotic pathways in BT549 and MDA-MB-157 cells.

## 5. Conclusions

Although the mechanisms and therapeutic effects of AG8 on TNBC need to be further investigated, the present results indicated that AG8 showed anticancer effects by inducing ROS, inhibiting ERK and Akt signaling pathways, and direct impairing the function of mitochondria. In addition, we found that different TNBC subtypes showed different sensitivities to AG8, and therefore, AG8 may have selective cytotoxic effects against AA and M TNBC subtypes. It is interesting that AG8 dramatically induced MDA-MB-157 (AA subtype) cell apoptosis through pathways not only associated with ROS, in contrast to the other two subtypes. The mechanisms of the effects of AG8 need to be further examined.

## Data Availability

The datasets generated during and/or analyzed during the current study are available from the corresponding author on reasonable request.
